# A Treatise on Neuralgia, or Painful Affections of the Nerves

**Published:** 1842-01

**Authors:** 


### ART. VII.

*Traité des Névralgies, ou Affections Douloureuses des Nerfs.* Par F. L. S. VALLEIX, Médecin du Bureau central des Hôpitaux, &c.—*Paris,* 1841. 8vo, pp. 719.

*A Treatise on Neuralgia, or Painful Affections of the Nerves.* By F. L. S. VALLEIX.—*Paris,* 1841.

IN the work, of which the following pages are an analysis, the author has carefully abstained from theorising, and has made few statements that are not borne out by the observations he records. The result has been a much greater degree of precision than is usually met with, and the ascertaining of several important points which have hitherto been generally overlooked. Still, though the treatise is in a great measure founded upon original observations, M. Valleix has not, like too many of his countrymen, neglected to avail himself of the labours of his predecessors of all nations, whenever they have been recorded with sufficient exactitude; but, as we shall perceive during our further progress, the number of these is much smaller than we might at first sight be inclined to suppose.

The following is M. Valleix's definition of neuralgia: "A more or less violent pain situated in the course of a nerve, and disseminated from circumscribed points; these points being the foci from which lancinating or other analogous pains proceed at variable intervals, and on which pressure is more or less painful." (p. 2.) This definition differs considerably from those in common use, and excludes all cases of what is called visceral neuralgia. Of its correctness, when applied to affections of the subcutaneous nerves, we can only judge after examining the symptoms more in detail; but we may shorly notice here the reasons which have induced our author to omit all consideration of those internal affections which are generally regarded as neuralgic. He argues that the symptoms, though in some respects analogous to those of neuralgia, differ from them in others which are no less essential. Thus, in visceralgia the seat of the pain occupies a considerable extent of the viscus, and cannot therefore be compared to the seat of ordinary superficial neuralgic pains. These visceral affections consist rather of functional disturbances of the disordered organs than of pains in the nerves which enter them; while in neuralgia properly so called, the pain of the nerve is the principal point, and the functional changes in the organs to which it is distributed are merely accessory phenomena. The nervous pains excited by cancer are indeterminate in their course, and do not present the same characters as those which are situated in a nervous trunk.

"I do not pretend," M. Valleix continues, "to say that these different pains have not numerous features in common, and do not approximate each other in their nature, but the difference of seat produces so great differences in their manifestation, progress, and appropriate treatment, that the practitioner is obliged to distinguish them carefully. In general pathology, it would perhaps be right to bring out their numerous affinities; in this work, in which facts are chiefly regarded in a practical point of view, I have thought it proper, on the contrary, to render their differences prominent. In other words, finding in the principal nervous trunks which ramify upon the surface of the body, an affection whose characters are clearly defined, I have formed a pathological group, upon which an exact and rigorous observation may be employed with every chance of success. This group is constituted by the NEURALGIÆ, distinguished from the *visceralgiæ* and *symptomatic nervous pains.*" (pp. 4-5.)

Whatever may be thought of the general arguments upon which this determination is founded, we are assured that M. Valleix has done wisely in acting upon it in the present instance, and that we are indebted to it for a much better book than would in all probability have been produced had he allowed himself to follow the more ordinary plan.

In proceeding with our analysis, we shall first take a general view of the subject, and then pass to the consideration of the different forms of neuralgia, reversing thus the order which our author has followed; and in doing this, we shall confine ourselves almost exclusively to those points which have been either omitted or differently represented by other writers, passing over, with little if any notice, such features of the disease as are generally recognized.

SITE. First, then, as to the seat of neuralgia. It is admitted on all hands that the superficial nerves of the body are of all others the most liable to these painful affections; but it appears that some points of their course claim a decided preeminence in this respect. According to M. Valleix's observations, these points are, with few exceptions, the following: 1. *The place of emergence of a nervous trunk*, as at the supraorbital and infraorbital foramina, where the branches of the fifth pair emerge upon the face; at the groin, where the crural nerve passes outwards; and at the lower part of the occipital bone, where the occipital nerve makes its exit. 2. *The points where a nervous twig traverses the muscles, to ramify in the integuments*, e. g., the passage of the posterior branches of the spinal nerves, &c. 3. *The points where the terminal branches of a nerve expand in the integuments*; for instance, the anterior extremity of the intercostal nerves, the extremity of the collateral nerves of the fingers, &c. To these may be added the situations where several branches of different nerves unite by their extremities, the most important of which is in the neighbourhood of the parietal protuberance, where the branches of the frontal and great occipital nerves are intermixed. 4. *The points where nervous trunks become superficial during their course*, as where the ulnar nerve turns round the internal condyle, and where the peroneal nerve turns round the head of the fibula. Of all these the points of emergence appear, upon the whole, the most liable to be affected, but in none of them can any normal structural variations be detected to account for this singular election.

CHARACTER. The nature of the pains in neuralgia is exceedingly various; but in the majority of cases two distinct kinds can be easily recognized, one dull, heavy, and constant, the other sharp, lancinating, and either irregular or intermittent. The attention of practitioners has been greatly limited to the latter of these; but if M. Valleix's observations be correct, the former is at least of equal importance, notwithstanding its comparatively less severe character. Cotugno held the same opinion, and regards it as the most essential element of sciatica, (p. 660.) It is generally compared by the patient to the sensation of tension, firm pressure, or the pain of a contusion, but is occasionally so slight that the patient will not allow its existence, until his attention is forcibly directed to it by his medical attendant. M. Valleix believes that it is never absent even in the most complete remissions of the attacks. It is most commonly found in the points noticed above, and these, our author affirms, are invariably, or with few exceptions, more or less morbidly *sensible to pressure.* As this opinion is decidedly opposed to the ordinary doctrine, and moreover as it forms the most characteristic feature of the work before us, we shall make no apology for examining it at some length.

If we consult the various authors who have written upon the subject of neuralgia, we shall find that their sentiments upon this point are far from being uniform. According to some, *slight* pressure upon the nerve aggravates the pain, while *firm* pressure relieves it; others believe that pressure is never painful; while, according to others, again, it is in rare cases only that pressure produces any effect. The former is perhaps the most generally received opinion; it is adopted by one of the most recent writers upon the subject of nervous diseases, to whose work we shall have occasion to refer more than once in the course of this article.* M. Valleix affirms that, *with one exception only*, (a case of sciatica which had only

* Romberg, Lehrbuch der Nervenkrankheiten, Band i.; Berlin, 1840.

existed from twenty-four to thirty-six hours, and was extremely slight, (p. 518,) he has in every instance succeeded in ascertaining the existence of more or less severe pain by pressure upon certain parts of the nerve. (p. 66.) This enormous proportion can scarcely be the effect of accident or mere coincidence; and the discrepancy between our author and other writers is susceptible, as we shall presently see, of a very different explanation. In some cases pressure merely increases the dull, constant pain, but in others it gives rise to violent twinges. A very slight touch is often sufficient to excite the most acute suffering, as many writers besides M. Valleix have borne witness, and as the experience of all of us testifies; while in other cases, the finger must be firmly pressed upon the part before any effect is produced. The *extent of surface* which presents this phenomenon is sometimes very small, not more than one or two *centimetres** in diameter, but occasionally it is much larger. At a short distance from these points, there is often a complete absence of pain under every degree of pressure, and this is doubtless one of the causes which have given rise to the great differences of opinions; for it can be readily conceived that painful points of so limited an extent may easily escape notice in a superficial examination, and also that pressure by the whole surface of the hand may produce no effect, because it will act in a great measure upon parts devoid of any unnatural sensibility. The only method, therefore, by which this essential point of diagnosis can be satisfactorily established, is by carefully pressing with the extremity of the finger along the entire course of the affected nerve, and of its principal ramifications, even though the patient should indicate a few points merely as the seat of his malady: for it is of importance to observe that, in some cases, there may be pain on pressure, where there is no spontaneous uneasiness, and *vice versâ*. The value of this sign will be abundantly evident as we proceed in our analysis.

It is necessary also to observe that, when the existence of acute pain in any particular spot has been ascertained, pressure in the same place, a short time afterwards, may fail in producing any effect; but this absence of pain will be found only of temporary duration. M. Basseau first noticed this fact with regard to intercostal neuralgia, and our author has been able to verify his observations. (p. 669.) It must, moreover, be always borne in mind, that there are some parts of the body, such as the anterior wall of the thorax, and the scrobiculus cordis, which are naturally painful under slight pressure in certain individuals, especially in those who are emaciated or of a nervous temperament; and in these cases an inattentive practitioner might easily be led into error. It is therefore necessary, before forming any opinion, to press with equal force upon the opposite side of the body. If the neuralgia be simple, and the affected side much more painful than the other, the inference will be easy; and even when the disease is double, there is generally sufficient difference between the intensity of the pain on the two sides, to establish this diagnosis; especially as in the majority of cases there will be found intervening spaces, in which pressure will produce little, if any suffering.

The pain caused by pressure generally bears a direct proportion to the intensity of the disease; it is also augmented during the paroxysms, and

* A centimetre is two fifths of an inch nearly.

diminished in the intervals of calm. It was asserted by M. Regnier and has been again more recently affirmed, that there is a great difference between the various species of neuralgia, in reference to the effects of pressure; that, for example, pain is almost constantly produced by pressing upon the affected nerve in sciatica, while in neuralgia of the face pressure often gives relief. M. Valleix has not observed this difference: in all the cases of neuralgia of the face which he has examined, he has found pressure give decided pain; but it must be remarked that the intermissions in this species are much more complete than in other neuralgiæ, and the pain excited by pressure often much slighter; and this may probably have given rise to the opinion. Everything, as he justly observes, depends upon the mode in which the pressure is applied. (p. 672.)

With regard to the lancinating pains our author observes, that in every case which he has examined, they have originated from one or other of the points occupied by the dull, constant pain above described, or in which pressure was painful. These, therefore, may be regarded as centres or *foci*, from which the pain shoots, with more or less violence and at variable intervals, to the neighbouring parts. Sometimes the pain darts from one point to another, without affecting the intermediate space; at other times it sets out at once from several foci. The following table (p. 662) shows the relative frequency of these different kinds:

| | |
|---|---|
| 1. Lancinating pains propagated from one point to another, without affecting the intervening course of the nerve . . | 9 |
| 2. Lancinating pains felt along the nerve . . . . . . . . | 34 |
| 3. Ditto fixed and disseminated . . . . . . . . . . | 16 |
| | 59 |

The direction of these lancinating pains is various; most commonly they follow the course of the nerve; sometimes they pass in the opposite direction, and when this is the case, the term *neuralgia ascendens* has been of late applied to the disease, though the fact was well known to Cotugno. Frequently the pains dart in all directions. The following varieties as to direction occurred in 109 cases: in 62 the lancinations followed the course of the nerve; in 16 they were fixed and disseminated; in 6 ascending; and in 11 they passed sometimes in one direction and sometimes in another; in 5 they proceeded both upwards and downwards from a middle point; in 6 they followed no determinate direction; and in 3 they did not exist at all. It would appear that upon the whole, the lancinations are most violent in neuralgia of the face and in sciatica.

*Course, duration, and termination of the disease.* The paroxysmal character of neuralgia is well established; Romberg regards it as the distinguishing feature of this class of diseases, (loc. cit., p. 12,) and M. Valleix has only met with one case in which the total absence of paroxysms was clearly ascertained; but opinions are still divided regarding the important question of the periodicity of the attacks. Out of 199 cases of all varieties of neuralgia, this symptom was satisfactorily made out in 20 only. It would seem that it is much more frequent in neuralgia of the face than in any other species, for our author has observed it in 10

out of 42 cases of that kind, and in 9 only out of 157 cases of the other varieties :* it is also well worthy of notice, that quinine effected a cure in about one half of these truly intermittent cases, while in others, precisely similar in every respect but this one, it totally failed. (p. 680.)

Much has been often said of the rapid accession of neuralgic affections. M. Valleix is of opinion that this only holds good with reference to the attacks of lancinating pain, for out of 71 cases in which this point was accurately noticed, in 38 the disease came on slowly, in 24 gradually but with greater rapidity, and in 9 only did it become suddenly violent; (one of these was very remarkable from the extreme obstinacy and violence of the disease. p. 115.) The invasion of the different parts of the nerve is also progressive, not instantaneous.

Sudden atmospherical changes are generally supposed to have a decided influence upon the intensity of the attacks; according to our author, depression of temperature has a remarkable effect in augmenting the violence of the pain, but other variations were apparently inoperative.

The *duration* of neuralgia is so essentially variable, that no mean term can be laid down. As to its *termination*, M. Valleix takes a more favorable view than the majority of practitioners, for he speaks of more than three fourths being radically cured. We cannot but think this is too sanguine an opinion, and the great liability of relapses ought assuredly to render our prognosis very guarded upon this point.

In regard to the essential *nature* of neuralgia, we are perfectly inclined to believe with our author, that the evidence we at present possess will warrant one opinion only, namely, that it is a functional affection of the nervous system, meaning by that term that its organic causes are not appreciable by any of our present means of investigation. The theories of inflammation, irritation, hypertrophy, &c. are totally devoid of anything like a satisfactory foundation; they have little value beyond that of affording amusement to the imaginative faculty.

CAUSES. Neuralgia is most frequent in the prime of life, but neither infancy nor old age are altogether exempt from it. M. Valleix relates a case of lumbar neuralgia in a girl of five years, and Dr. Rowland has given the history of another aged two years only, who was affected with facial neuralgia. (Rowland on Neuralgia, p. 12.) In a table drawn up by our author, the three decades included between the ages of twenty and fifty present almost equal numbers, including nearly two thirds of the whole (296 cases.) Opinions are strangely divided with reference to the comparative liability of the two sexes; Fothergill and others maintaining that females are much more frequently affected, while Thouret, who examined the subject with much care, affirms that two thirds of the whole number of cases are of the male sex. M. Valleix believes that, as a general rule, both sexes are almost equally liable, the female preponderating in a very slight degree, but he has shown that there is considerable variety when the different species of neuralgia are examined. Thus in cases of sciatica and crural neuralgia, the proportion of males greatly exceeds that of females, while precisely the opposite obtains in the intercostal and lumbo-abdominal varieties, (Vide table, p. 691.) He

* These are the exact numbers given in the original; there is evidently a mistake in one of the statements, probably in the former.

has also ascertained that before the age of thirty, females are more liable to the disease than males, but the tendency is rather the other way afterwards. These results of course require confirmation from the examination of larger numbers, before the point can be regarded as satisfactorily determined; we give them here in order that the attention of enquirers may be more closely directed to the subject.

Our author's observations on the influence of constitution, temperament, hygienic conditions, food, and professions are too limited to be of much value; but all these points deserve more notice than they have yet obtained. Of all *exciting* causes, M. Valleix believes that prolonged exposure to cold is the most frequent, but this portion of his work is extremely meager, and we shall therefore pass on to the article

Diagnosis. Neuralgia may be confounded with other affections seated in the nerves; the most important of these is undoubtedly *neuritis.* The distinctive marks generally laid down between the two affections are the following: in neuritis the seat of the pain is more fixed, the duration of the disease is shorter, and the remissions less pronounced; the lancinating pain returns more gradually, there is more tendency to paralysis, and the pain is increased by pressure. M. Valleix believes, that, with the single exception of the greater tendency to complete paralysis, all the other symptoms are common to the two affections, and in this he appears to be borne out by the history of a very interesting case, in which the sciatic nerve was injured during a severe labour, where the forceps were required to complete the delivery: but still he leaves the question very much in doubt. Unless all the observations recorded in this book be incorrect, pain on pressure is not a distinctive mark, since it equally exists in pure neuralgia; but we would suggest, that probably a further examination may show that in neuritis the pain excited in this way may not be so remarkably circumscribed; it would seem more natural that the whole extent of the affected part of the nerve should present the same symptom. In this opinion we are confirmed by the observations of Professor Romberg, who remarks that, in the affection of which we are speaking, there is severe pain *along the whole course of a nerve*, which is increased by external pressure or motion. He adds, that when the nerve is subcutaneous it can be felt to be hard and enlarged, and that as the disease advances, *anæsthæsia* is gradually produced, while neuralgia, on the contrary, is characterized by the perfect uniformity of the symptoms throughout the entire course of the malady. (Loc. cit. pp. 16 and 29.) When the disease is acute the existence of fever will assist the diagnosis.

*Pricking*, *laceration*, and *contusion of nerves*, give rise to lancinating pains, which closely resemble those of ordinary neuralgia, and are generally regarded as such. M. Valleix expresses himself as unable to give a decided opinion from the want of sufficiently detailed observations; and applies the same remark to certain cases of neuroma, and to those instances of violent pain after amputations, which appear to depend upon a diseased condition of the extremities of the divided nerves. We would take the opportunity of observing here, that it appears to us a most unhappy tendency, which is evidently gaining ground, to describe all severe pains situated in the course of a nerve by the common term *neuralgic;* such an appellation is undoubtedly correct enough in a mere etymological point of view, but if it be true that the disease denominated neuralgia has certain specific characters, it is manifestly unadvisable to employ the same name where the existence of these has not been established.

Neuralgia may also be confounded with *rheumatism*, but in general it can be pretty easily distinguished by the greater extent of surface affected in the latter disease, and by the great increase of pain caused by contractions of the muscles. In cases of acute articular rheumatism there can be no room for doubt.—We pass over all other diseases which may be mistaken for neuralgia, because they will be more profitably noticed in considering the various species of this painful affection, to which we shall now proceed.

TRIFACIAL NEURALGIA. M. Valleix commences his description of this variety by a sketch of its history, of which we shall take no further notice than simply to remark that it has the merits of being full, concise, and impartial; nor shall we delay our progress by dwelling upon the anatomical distribution of the fifth pair of nerves. The number of cases analysed by M. Valleix is fifty-five, twelve of which are original, and the others borrowed from various authors. In speaking of these, our author makes a remark, the truth of which will be readily admitted by all who have at any time attempted to deduce general principles from recorded observations:

"It is sufficient to study the history of neuralgia, in order to be convinced how unfounded is the general opinion of the richness of our science in regard to observations. Facts are doubtless abundant, and by searching various authors, and especially periodical publications, one may collect a large number; but what is the value of mutilated facts, deficient in the most necessary details, and very often presented for the purpose of establishing a theory? We are not therefore wrong in crying out for new facts every day; but it must be understood, that what is required is not such records as the majority of our predecessors have left behind them, but facts observed with care and strictness, by which alone the science may be advanced." (pp. 20-1.)

M. Valleix believes that neuralgia of the face is less common than the sciatic dorso-intercostal varieties, in which opinion he differs from other authors: it is not easy to say with whom the truth lies. It is somewhat more common with females than with males, but the difference is extremely slight. In most of the cases examined by our author, there was more or less disturbance of the menstrual functions, but this often supervened upon the disease, and therefore could not be regarded as a cause. He has never known the disease have either a metastatic or a syphilitic origin; nor has he met with any case produced by external violence, or dependent upon caries of the teeth, though both of these are well known causes. Both sides of the face seem equally liable to be affected, but the pain is often most severe when seated on the right. Of forty-six cases, twelve only were double. Two kinds of pain may be distinguished in this as in the other species of neuralgia, one dull and constant, the other lancinating. M. Valleix finds that the first does not occupy the entire course of the nerves, but is disseminated in different points of the three principal branches, in the following manner.

A. *In the course of the ophthalmic branch.* 1. At the *supra-orbital foramen.* In eleven cases out of fourteen there was constant pain in this locality over an extent of surface which, in all but two instances, did not exceed two or three centimetres. In two other cases the seat of the pain was a little above the foramen, but still in the course of the frontal nerve. M. Valleix denominates this the *supra-orbital point*. 2. In two subjects pain existed in a more undefined situation along the rim of the orbit, and in the upper eyelid. Three cases are mentioned from other authors, in which the same is vaguely noticed. M. Valleix calls this the *palpebral point*, and believes that the palpebral twig of the nasal branch is the part chiefly affected. 3. In three subjects there was pretty severe pain in a circumscribed spot at the upper part of the side of the nose, where the infra-trochlear nerve emerges from the orbit. This *nasal* point is noticed, but not defined, in sixteen cases drawn from various sources. 4. Several of M. Valleix's patients complained more or less of pain in the ball of the eye; one of these was not affected in any of the other points mentioned. In eight cases related by different authors the same fact is noticed, and in five of these the other portions of the ophthalmic branch were not the seat of any suffering. M. Valleix observes, that he has never been able to satisfy himself of the existence of pain in the nerve while inclosed in its bony canal, or lying in the interior of the orbit; it is not attacked by the disease until it becomes superficial.

B. *In the course of the superior maxillary branch.* 1. At the *infra-orbital foramen.* Pain was only observed in this point in three cases out of fourteen; it is therefore of less importance than the preceding. M. V. has only found it mentioned six times by other authors. This point varies in diameter from four to five centimetres. 2. *On the cheek.* In two original cases, and in four borrowed ones, a painful point is noticed on the lower edge of the *malar bone.* 3. In seven out of sixteen cases the gums and teeth of the upper jaw exclusively were complained of by the patient as being the seat of constant pain, and in nine others both jaws were affected alike. 4. M. Valleix has never found the upper lip particularly painful, but mentions two examples from other authors. This *labial point* he considers of little importance. 5. The same remark applies to pain in the *palate ;* four remarkable examples of which are however noticed by other writers.

C. *In the course of the inferior maxillary branch.* 1. On the *temple.* In six cases tolerable severe pain was felt in a circumscribed point, not more than two centimetres in diameter, at the lower part of the temporal region, a little in front of the ear. This *temporal point* deserves particular notice. Several nervous branches ramify here, but the largest and most superficial is the temporal branch of the inferior maxillary nerve; M. Valleix regards it therefore as the seat of the affection, both from general reasoning, and from the fact that in a patient under his care at the time he was writing, he finds a painful spot immediately in front of the tragus, at the precise point where this nerve emerges from the parotid gland. 2. In one subject only has he found a painful spot in the situation of the *temporo-maxillary articulation.* 3. In five cases M. Valleix has discovered the existence of pain in a spot about two centimetres in diameter, in the situation of the *mental foramen,* and in all these the *temporal point* above noticed was very distinct: a corroborative proof of the correctness of his opinion regarding the former. 4. In one of M. Valleix's cases the side of the *tongue* was painful, and four other cases are on record. In all these the disease was of a very violent character. 5. In the case just mentioned there was also pain in the *lower lip*, near the foramen menti.

D. *In the points where the branches of the trigemini anastomose.* Several of the points above noticed, as the *nasal* and the *labial*, might be referred to this division; but M. Valleix remarks that, in the face generally, the points where the nerves emerge from deep-seated parts to ramify in the skin are more severely affected than their ultimate extremities; the contrary, however, is the case in regard to the point which we are now to consider, and which our author terms the *parietal point*. It is most commonly situated a little above the parietal protuberance, and varies in extent from three centimetres upwards. He has met with it twelve times, and believes that it will always be found to exist when the disease has any degree of violence, unless the inferior dental nerve be alone affected. It is situated at the junction of the frontal, superficial temporal, and suboccipital nerves. This point has been noticed by other authors, but its extent not defined.

Our author next proceeds to examine the interesting question of the dissemination of the disease, and with the following results:

| | | |
|---|---|---|
| Painful points | in all the three branches . | 24 times. |
| „ | in two branches only . | 11 „ |
| „ | confined to one branch . | 10 „ |
| | | 45 |

It appears, therefore, that it is much more common to find the affection seated at the same time in several parts of the trifacial nerve, than limited to one division only. When the latter was the case, the inferior dental nerve was most frequently the diseased part. In point of fact, therefore, Chaussier's minute subdivisions are of little practical importance.

As to the point first attacked, M. Valleix remarks that out of thirteen cases the pain commenced at the supraorbital foramen, on the side of the nose, or in the upper eyelid, in eleven, and twice in the course of the inferior dental nerve, where it remained fixed. He has never known the disease originate in any branch of the superior maxillary nerve. In examining the cases recorded by other writers, he finds only nine presenting sufficient details to establish this point, and in five of these the affection first made its appearance in one of the branches of the ophthalmic, while the remaining four commenced in and were limited to the inferior dental. Professor Romberg, on the contrary, affirms that the superior maxillary is the most frequently affected, and next to that the ophthalmic, but he does not give any numerical statements. (Loc. cit. p. 34.)

We proceed now to the consideration of another most important symptom presented by these foci. *In all the cases* which have come under M. Valleix's own observation, pressure, properly exercised by the finger, was productive of pain in one or more of the points above noticed. It is to be lamented that the number of these cases is so small, only fourteen; but the undeviating uniformity presented by them is certainly very striking, and calls for more careful attention on the part of succeeding investigators. Out of thirty-two cases recorded by other practitioners M. Valleix has found fifteen in which the same symptom was noted, and it is a remarkable circumstance, and one strongly corroborative of the correctness of the view taken by our author, that the more detailed and exact the observations are, the more frequently has this sign been detected. Such writers as are contented with the simple assertion that the pain occupied one side of the face, &c., will never add much to our knowledge of this most troublesome malady. M. Valleix believes that such cases as do not present the symptom now under consideration must be regarded as mere exceptions: we are inclined to recommend a much more extended series of observations before laying down so general a rule. The narration of the case which follows these remarks (p. 68 et seq.) is exceedingly interesting, and we would recommend the style in which it is drawn up as a model for future enquirers; but it is much too long to be transcribed, and we shall content ourselves with the notice of one feature which is peculiarly worthy of attention. The unfortunate sufferer, during the paroxysm of pain, was in the habit of pressing her open hand firmly upon the left side of the head, and appeared to receive comfort from it, but the application of the extremity of the finger to the point where the frontal nerve emerges immediately increased her suffering, and *the pain became intolerable when the part was firmly pressed.* This fact speaks volumes; an equable pressure over the whole surface gave great relief, while in one small spot, only a centimetre in diameter, the contact of the finger, even when *firmly* applied, was perfectly unendurable. Have we not here an explanation of the discordant results of former investigations?

Nor is it merely in a diagnostic point of view that this sign is valuable. M. Valleix affirms that as long as pressure upon any of these points continues painful, there is great danger of a speedy recurrence of the attacks; it is the last symptom which gives way, and the cure may be regarded as complete (for the time ?), when the finger can be applied to them all without producing more discomfort than it always does in the situation of even the most healthy nerve.

In the majority of instances the seat of this artificially produced suffering corresponds with that of the constant spontaneous pain; but this rule is liable to a few exceptions, as will appear by the following statement. Out of thirty-five cases, in twenty-six the two species of pain had the same locality; in four there was pain on pressure where no spontaneous pain existed; and in five the contrary obtained. It should be remarked, however, that all these exceptions are found in the cases recorded by other authors: in M. Valleix's own observations the two kinds were invariably coexistent.*

In the description of the pain produced by motion, mastication, deglutition, &c., we find nothing peculiar. M. Valleix has only met with one case of facial neuralgia in which lancinations were entirely wanting; but not all his patients experienced them at the commencement of the disease, and in several they ceased before the cure was effected: he therefore does not regard them as absolutely essential symptoms. (p. 83.)

We pass unnoticed the other anomalous feelings which are so fre-

* From a slight *personal* experience of this form of neuralgia, we are enabled to bear testimony to the existence of the supraorbital, nasal, malar, and (less distinctly) the mental points. And we can also affirm the correctness of M. Valleix's statement that these points are most painful upon pressure, when the spontaneous pain is greatest.

quently the subjects of complaint in neuralgic patients. The state of the eyes, ears, nasal passages, &c., deserve attention on the part of practitioners, but there is nothing in our author's account which need detain us. In five cases the bulbs of the hairs were the seat of acute pain, and a remarkable case of supraorbital neuralgia of the left side is quoted from Bellingeri, in which the hairs of all the anterior part of the affected side became more bristly, and thicker, with a rapid growth. The disease was cured by section, and subsequent cauterization of the nerve, and then the hairs again returned to their normal condition. The same author mentions the occurrence of crepitation on the affected side of the head. M. Valleix has seen two cases in which abundant *salivation* was produced: André affirms that he always met with it.

Our author next proceeds to examine the importance of *convulsions* or *spasms of the face* as diagnostic signs, and comes to the conclusion that they cannot be regarded as essential; in which opinion we believe the majority of observers will be fully inclined to coincide; but to this point we shall have occasion again to recur.

The affection we are now considering is frequently complicated with other species of neuralgia, the occurrence of some of which may probably be accounted for by the existence of nervous anastomoses while in others this explanation will not hold. M. Valleix has never seen the disease *suddenly* transferred from the face to another part of the body, but undoubted instances of this nature are to be found in the annals of medicine, and are of no little interest. His remarks upon the state of the circulatory, digestive functions, &c., need not detain us. He only met with four cases in his own practice in which periodicity was well marked, but mentions that Dr. Renner, in the space of fifteen months, attended thirty-two cases of facial neuralgia, all of which presented a more or less distinctly periodic character, and yielded to the influence of sulphate of quinine. Romberg states that periodicity is most frequently observed when the supraorbital nerve is affected, that it is generally of the quotidian type, less commonly of the tertian, and never of the quartan. He asserts also that the disease is generally of shorter duration when this symptom is observed. (Loc. cit. p. 36.) According to M. Valleix's observations it is not correct to assert, as was done by Frank, that the paroxysms are more common during the night than the day. It is of importance also to bear in mind, that in the intervals of the exacerbations there is generally still a dull pain in the points above noticed; a *perfect* intermission is regarded by our author as a very rare exception.

We shall say nothing of the anatomical lesion occasionally observed; M. Valleix has made no addition to our previous knowledge, vague and unsatisfactory as that is. In regard to the *causes* of the disease we find nothing peculiar in the work before us; the subject is involved in great obscurity, and demands much careful observation before any positive assertions can be hazarded.

DIAGNOSIS. Our author believes that nervous headach (migraine) may be distinguished from neuralgia by its shorter duration: we can scarcely regard this as a sufficiently diagnostic mark, and are much inclined to believe that, in many cases, the seat of the affection is the superficial nerves of the scalp. *Clavus hystericus* is regarded by M. Valleix as a species of neuralgia.

We now arrive at a question of no little physiological interest. *Can the portio dura of the seventh pair be ever affected with neuralgia?* Considering the functions of this nerve, one might be readily induced to answer at once in the negative; but, as M. Valleix well remarks, it does not necessarily follow that, because it is physiologically insensible, it must continue the same when affected with disease: we shall, therefore, give a short abstract of the opinions entertained by various writers. In doing this, however, we shall not enter into the discussion whether the marks of ordinary sensibility exhibited by some portions of the nerve are or are not due to its intimate connexion with the ramifications of the fifth pair, for there are few, we apprehend, in this country at least, who have any doubts whatever upon the subject. The majority of the descriptions of this supposed affection of the portio dura are sufficiently vague: some observers inform us that the pain extends along the course of the nerve, commencing at its point of emergence; others merely affirm that it *appears* to follow this course; while others again are contented with saying that they have seen cases of neuralgia of the facial nerve! What confidence can be rationally placed upon such loose and uncircumstantial notices? M. Reverdit, however, goes much farther than any others, for he recognizes three varieties of this affection. In the first of these, the pain commences towards the back of the auricle, extends to the temple, the cheeks, the nose and the lips, and *appears more superficial than in cases of trifacial neuralgia;* in the second, the pain passes in one direction towards the chin and neck, and in the other stretches from the auricle to the shoulder; in the third, it extends to the mastoid region and occipital bone. Now, with reference to these assertions, there is an observation which will naturally strike the reader, namely, that as the extremities of the facial nerve and the fifth pair occupy the same localities, it does not follow that pain seated in the temple, cheek, chin, &c., must of necessity be referred to the former. As to the pain in this affection being more superficial, any one who has paid the least attention to the descriptions of their ailments given by patients, will know at once how much value there is in this declaration. The only statement, therefore, of any real importance is that which refers to the point where the pain commences, but the difficulty will be greatly removed even in this case, if we recollect, that a large nerve, the posterior branch of the second cervical pair, passes close by this point to ramify in the hairy scalp, where its branches anastomose with divisions of the fifth pair. M. Bérard the elder, relates the case of a young man with disease of the cervical vertebræ, who suffered from intolerable pain in the back of the head, and after death a reddish gangliform tumour was found at the origin of this nerve, which had evidently been the cause of these symptoms. Several cases are also quoted by our author in which the pains commenced precisely in the situation of this posterior branch, and then radiated to different parts of the face, (p. 163:) it is impossible to quote them here, without occupying a greater space than we can afford, but we would earnestly recommend their attentive perusal. M. Valleix remarks that in many of the instances of this kind which he has observed, (and which he includes under the term *cervico-occipital neuralgia,* to be presently mentioned,) the patients invariably informed him at first that the pain commenced behind the ear, and passed from thence to the face; but a more close investigation, especially by means of pressure, convinced him, 1, That the point of origin was not immediately behind the ear, but between the mastoid process and the vertebral column; 2, That the lancinations passed from that spot upon the occipital bone, and reappeared at the parietal protuberance; and 3, That in this place there was a painful point, from which lancinations radiated to different parts of the face, and gave rise to all the ordinary symptoms of *tic douloureux.*

This must conclude our notice of neuralgia of the face; we defer the subject of treatment until we have reviewed the other species, when we shall have the advantage of considering the whole at once.

CERVICO-OCCIPITAL NEURALGIA. The existence of this variety had been suspected by several observers, before M. Bérard gave an accurate delineation of the symptoms, still retaining, however, the name tic douloureux. The nervous branches concerned in this affection are those which belong to the posterior cervical plexus, especially the posterior branch of the second pair, called by Arnold the *great occipital nerve;* and several branches of the anterior cervical plexus, as the *superficial cervical*, the *auricular*, the *mastoid* or *small occipital* of Arnold, and the *supra-clavicular* and *acromial.* In this species of neuralgia, which is of rare occurrence, the lancinations ordinarily commence from a point at the lower part of the occipital bone between the ear and the spine, and pass either upwards over the vertex towards the face, in which case the fifth pair generally becomes involved, or downwards along the neck to the shoulder. Sometimes they are arrested at the parietal protuberance; and occasionally, instead of commencing as above described, they set out from the upper part of the mastoid process, or from the parietal protuberance. The following are the points in which pressure developes pain: 1, and most severely, at the emergence of the *great occipital nerve;* 2, lower down, between the anterior edge of the trapezius and the posterior edge of the sterno-cleido-mastoideus, where the principal branches which form the *superficial cervical plexus* make their exit; 3, the *parietal point* noticed before when speaking of facial neuralgia; 4, on the *mastoid process* below the lobule of the ear; 5, lastly, an *auricular point* upon the concha. These different points are not always so accurately defined as in the other species, but this is easily accounted for by the mode in which the nerves are distributed.

CERVICO-BRACHIAL NEURALGIA. Under this head M. Valleix includes every neuralgia which is seated in the brachial plexus, and in the posterior branches of the last cervical pairs, whatever nerve may be more especially affected. We shall not delay our progress by describing the anatomy of the parts, but content ourselves with calling the following facts to the reader's recollection: 1. That the nerves which form the brachial plexus give off a series of posterior branches, which traverse the muscles, and enter the skin near the spine at the lower part of the neck. 2. That the brachial plexus becomes superficial in the axilla. 3. That before furnishing its terminal branches, the plexus gives off the *supra-scapular* and *circumflex* nerves, the former of which is remarkable from its position upon the spine of the scapula, and the latter for its course round the surgical neck of the humerus, and for the cutaneous branches which traverse the deltoid. 4. That the *internal cutaneous* nerve, which is superficial for a considerable distance along the inner part of the arm, supplies the integuments over the internal condyle, and the anterior part of the wrist, and also gives off a filament which often passes in front of the median basilic vein, and may be injured in venesection. 5. The *median nerve* is chiefly interesting where it passes through the pronator teres, where it gives off the palmar cutaneous nerve, and in its terminal branches along the sides of the fingers. 6. The chief points to be observed in the *ulnar nerve* are its positions behind the internal condyle, its passage between the piriform and unciform bones, and its distribution to the sides of the three last fingers. 7. In the *radial nerve* we have to remember its course round the humerus, and the cutaneous branches which it gives off at its entrance into and exit from the humeral groove.

From so extensive a distribution of nerves it may be readily anticipated that the parts affected by the pain must vary considerably; such indeed is the case, and hence pathologists have divided the species into a number of varieties; M. Valleix objects to this method, because he believes that in most instances, even when the disease appears decidedly localized, a more attentive examination would reveal the existence of pain in other branches than those chiefly affected. The subject appears to us of little moment, it is rather a question of convenience than anything else.

Out of eleven cases the disease was situated on the left side in seven. M. Valleix has never found it double.

Points painful on pressure are as easily recognized in this as in other species of neuralgia, and of these the following are the most remarkable: 1, an *axillary* point; 2, an *epitrochlear*, where the ulnar nerve turns round the bone; 3, a *cubito-carpal*, where the same nerve passes in front of the carpus to the hand; 4, where the radial nerve turns round the humerus, and another on the same nerve towards the lower part of the radius; 5, an *inferior cervical* point at the lower part of the neck, a little to the outside of the cervical vertebræ; 6, a *post-clavicular* point within the angle formed by the clavicle and acromion; 7, a *deltoid* or *circumflex* point at the middle and upper part of the deltoid. The number of these which are affected will, of course, vary in different cases; the most circumscribed are those behind the internal condyle, and in front of the lower part of the ulna and radius.

M. Valleix's experience as to the nerve which is most frequently the seat of lancinating pains is in perfect accordance with the results of former observations. There can be no doubt that this preeminence is justly claimed by the *ulnar*. It is needless to describe the course of the shootings. We would again direct the reader's attention to the very interesting cases which our author has collected.

DORSO-INTERCOSTAL NEURALGIA. Nicod in 1818 first called the attention of practitioners to this species, the existence of which had, however, been previously suspected by Chaussier; since then it has been frequently noticed, and described under a variety of names, which we need not mention. M. Valleix has had the opportunity of examining a considerable number of cases of this affection; the following account of the disease is founded upon the analysis of 25, the only ones which presented sufficiently enlarged details.

In reference to the anatomy of the intercostal nerves, we have to bear in mind, that each nerve gives off an *anterior perforating branch* near the sternum, and a *middle perforating branch* at the middle of the intercostal space; and also that the *posterior branches* for the spinal muscles send some filaments through them, which ramify in the skin; these may be called the *posterior perforating branches.*

Our author regards this as a very common affection; he has met with it in subjects of an advanced age, and in one infant, but the interval between seventeen and forty years presents the greatest number of cases. The majority of patients were females. The left side of the chest is more frequently affected than the right, and the sixth, seventh, and eighth spaces seem peculiarly liable to the affection. The most interesting point ascertained by M. Valleix, is undoubtedly the existence of three points in the course of the nerves which are painful upon pressure. The first of these is situated a little to the outside of the spinous processes, almost opposite the exit of the nerve from the intervertebral foramen; this is the *posterior* or *vertebral point;* the second is at the middle of the intercostal space, the *lateral point;* while the third will be found between the cartilages, a little to the outside of the sternum, or in the epigastrium a little to the outside of the median line,—this is denominated the *anterior, sternal* or *epigastric point.* All these had a very circumscribed extent, and beyond them pressure could be firmly exerted without producing any particular discomfort to the patient. M. Valleix has never found the posterior point wanting; the anterior existed in 19 out of the 25 cases, and the lateral in 17. The exact position of the anterior is more variable than that of either of the others, and in some instances it was found to be multiple.

On the lancinating and dull constant pains it is not necessary to make any remarks: they are often increased by forcible inspirations, coughing, movements of the arms, &c. It is worthy of notice, that M. Valleix could never ascertain any relation between exacerbations of pain in the epigastric point, and derangement of the stomach; and that in only one case were the intermissions regularly periodic. M. Bassereau is of opinion that this affection is invariably connected with uterine disorders; our author's experience leads him to believe that there is little difference in this respect between it and other varieties of neuralgia, and that the most remarkable circumstance noticed by that observer, namely, the existence of a painful spot upon the cervix uteri, which will be more particularly considered immediately, was a symptom of a complication with another species of neuralgia, the *lumbo-abdominal.* By attending carefully to the circumstances noticed above, the diagnosis, which is often found so puzzling in painful affections of the thorax, will be greatly facilitated. The existence or absence of disease in the respiratory organs must of course be carefully ascertained by means of auscultation and percussion. The want of that peculiar anxiety and sense of impending dissolution, which is so characteristic of angina pectoris, distinguishes the affection we are now considering from that more important malady. It is more difficult to lay down rules for discriminating it from rheumatism of the thoracic parietes, but in general the seat of the pain in the latter affection will be found less circumscribed, and the three points not distinguishable. The suffering is also greatest when the muscles are called into action. For other differential symptoms we must refer to the original work itself. (pp. 408-11.)

In acute diseases of the spine there can be no difficulty in making the diagnosis. Chronic diseases are more liable to be mistaken, but even in these M. Valleix believes that it will be generally found that pressure is more painful immediately over the spinous processes than at the sides, while precisely the contrary obtains in this neuralgia. Our author is of opinion that the affection known in this country and in America by the name of *spinal irritation* is nothing more than neuralgia: on this point we shall not hazard an opinion, but shall merely quote the following observation as worthy of attention:

"The pain produced by pressure is situated at the exit of the nerve, on a level with the intervertebral foramen, and in no other position; now, if there had been really an irritation of the medulla, and if this irritation were the cause of the disease, in some cases, if not in all, the pain should be found at the origin of the nerve, and consequently in a point more or less elevated above the intervertebral foramen, in proportion as the neuralgia is seated lower down. But this will be seen not to be the case." (p. 345 )

Intercostal neuralgia is, perhaps, the most important variety, in a practical point of view, hitherto noticed. It is extremely frequent in this country, and, according to our experience, almost confined to young women: it is constantly mistaken by practitioners for chronic inflammation of the thoracic and abdominal organs, especially of the pleura, lungs, liver, and spleen, to the great injury of the patients.

LUMBO-ABDOMINAL NEURALGIA. This variety of the disease is still less known than the preceding, the only form under which it has been studied, being that which is denominated *ileo-scrotal neuralgia.* In reference to the anatomy of the parts concerned, it is necessary that the reader should bear in mind, 1, that the posterior branches of the lumbar nerves become subcutaneous at the outside of the spinous processes; 2, that there are several pretty large cutaneous filaments which run downwards to the integuments of the hip, and intersect the crest of the ilium at right angles, in front of the common lumbar mass of muscles; 3, that the cutaneous filaments of the abdominal branches of the lumbar plexus terminate in the skin over the lower part of the rectus; 4, that the anterior cutaneous branch of the external inguinal nerve becomes superficial at its exit from the femoral arch; and 5, that the scrotal nerve terminates in the skin of the scrotum of the male, and of the labia majora in the female.

Like intercostal neuralgia, with which it often coexists, this affection is more commonly seated on the left than on the right side; but of thirteen cases, it was situated eight times on the left; the other five were double, and of these, in two the pain was most severe on the right, and in three on the left side. (p. 453.) The first pair of lumbar nerves are most involved in the disease. Sometimes the pain is limited to the posterior muscles, and at others the anterior also participate; the symptoms of course vary accordingly.

The following are the points which exhibit pain upon pressure: 1, a *lumbar point*, a little to the outside of the vertebræ; 2, an *iliac point*, a little above the middle of the crest of the ilium; 3, a *hypogastric point*, above the inguinal ring, and to the outside of the linea alba; 4, an *inguinal point*, towards the middle of Poupart's ligament; 5, a *scrotal point*, towards the lower part of the testis, in the labium. Of these the hypogastric is the one most frequently wanting. A reference to the anatomical facts above noticed will explain the exact localities.

It is in this variety that the painful point upon the cervix uteri, which is so much insisted on by M. Bassereau, is most uniformly met with. We quote the following case, because the symptoms are well marked, and the non-existence of any complications relieves the diagnosis from all doubt:

CASE XLI. "A young laundress, aged 17, unmarried, stout, of ruddy complexion, with the pilous system slightly developed, presented herself at the Bureau Central, December 8, 1840. She has menstruated for a year. Six months ago the catamenia were suppressed for three months, and at the same time an abscess formed at the base of the jaw. Since then she has been perfectly regular. She lives in a warm, light, and dry room. Eight days ago, without any known cause, she perceived a pain, which commenced at the lower part of the abdomen, and afterwards extended to the loins. Pressure is painful over a space of four centimetres to the outside of the lumbar spinous processes, as far as the sacral region, on the right side; along the crest of the ilium to the extent of two centimetres; and in the hypogastrium over a space of about four centimetres. There is a similar pain, but to a much slighter extent, in the corresponding points of the left side. Pressure is not productive of any pain in the intervals between these points. The cervix uteri is firm and small, extremely painful to the touch on the right side, much less so on the left. No discharges, and no other pains. Little appetite; considerable thirst; bowels regular; no nausea nor vomiting. Pulse 92, soft and regular, with no abnormal character." (p. 450.)

In this case it will be observed, that the most painful side of the cervix uteri corresponded with that in which the other neuralgic symptoms were the most prominently exhibited; there were no evidences of organic disease or inflammatory action in any of the parts, and the natural inference is therefore that the whole depended upon the same cause, viz. a functional affection of the different nervous branches. In some of the other observations related by our author, the coexistence of leucorrhœa, blennorrhagia, &c. might throw some doubts upon the true nature of the case; but supported as they are by this, in which no such chances of fallacy can be detected, it appears scarcely possible that the diagnosis should be incorrect. We refer to the work for further information upon this remarkable point, and for the arguments by which M. Valleix combats the opinions of M. Bassereau, that the pain is the result of inflammation. (p. 403 et seq.) We do not conceive that there can be any difficulty in distinguishing between this affection and lumbago, gravel, &c. if the practitioner be sufficiently careful and minute in his enquiries. M. Valleix gives the differential marks, but we have not room to transcribe them.

CRURAL NEURALGIA. This is an extremely rare variety, excepting as a complication of sciatica. It was known to Cotugno, and is introduced into Chaussier's synopsis under the name *neuralgia fémoro-prétibiale.* Our author has met with two cases of the uncomplicated affection, and seventeen in which it was combined with sciatica. The crural nerve and its branches are the parts concerned. The following abstract of one of the two cases will give the best idea of this variety.

CASE XLVI. *Crural neuralgia of the right side.* "A young man, aged 23, a jeweller, presented himself at the Bureau Central, December 10, 1840. ....

Three weeks ago he was suddenly seized in the morning when rising, with a severe pain in the right groin, which gradually passed downwards to the dorsum of the foot. Lancinations were soon observed in the groin, and almost immediately afterwards in the foot, without affecting the rest of the limb; in other words, there were two consecutive lancinations, one in the groin and the other on the dorsum of the foot, but the pain passed over the interval between these two points, without manifesting itself in the course of the nervous filaments. These lancinations still exist, and are tolerably frequent. They sometimes pass to the nates, but never to the loins. When they are absent the patient is conscious of a sort of numbness, and a dull fixed pain in the parts above indicated. Pressure is painful in several circumscribed points, viz. in the groin, at the external and superior, the middle, and the internal and inferior aspects of the thigh, and above the knee. There are also two equally remarkable points, in part of the internal malleolus, and at the base of the first toes. The most extended of these is not more than four centimetres in diameter, and the intervals are perfectly insensible to pressure. The patient has severe pain when walking, chiefly when the foot is placed to the ground." (p. 471.)

This case presents all the points, painful on pressure, that our author has detected, excepting one, which is on the inner side of the sole of the foot. It is also an example of a phenomenon, which is by no means uncommon, though difficult enough to be explained, viz. the simultaneous appearance of pain at the two extremities of a nerve, the interval remaining perfectly free.

Femoro-popliteal neuralgia.—Sciatica. In pursuance of an original design, we shall in a great measure confine ourselves to the notice of such points as have not been prominently brought forward by other observers: the affection itself is too well known, and has been too frequently described, to require, or even admit of any lengthened account.

For the compilation of this article, M. Valleix has analysed 125 cases; but of this large number 36 only were complete in all their details; 15 of these 36 are original, the remaining 21 are borrowed from M. Louis. In reference to the anatomy of the parts concerned, we would recall the following points to the reader's recollection: 1, the series of *posterior branches* of the several nerves ramifying in the skin over the posterior attachments of the gluteus maximus; 2, the exit of the *gluteal nerves* through the upper part of the sciatic notch; 3, the *ascending* branches of the superior gluteal, which run as far as the crest of the ilium; 4, the *crural branch* (*posterior middle cutaneous*) of the inferior gluteal, which furnishes a number of filaments to the skin along the biceps; 5, the course of the *external popliteal sciatic*, (*popliteal* and *posterior tibial*,) which approaches the skin in the popliteal spaces, and becomes still more superficial at the head of the fibula, round the neck of which bone it turns, and which gives off the *external malleolar nerve*, some *dorsal branches* and *collateral nerves of the toes*; 6, the *external saphenous nerve*, which becomes superficial at the middle and lower part of the calf, passes to the outside of the tendo achillis, then behind the internal malleolus, and gives origin to the external calcaneal nerves; 7, lastly, the *lower part of the tibial nerve* which descends along the inner side of the tendo achillis, passes behind the internal malleolus, and divides into two plantar branches. It should also be remembered that these branches frequently communicate with each other, for this has an important influence on the dissemination of the symptoms.

This form of neuralgia is one of the most common; as we have already observed, M. Valleix conceives that in point of frequency it ranks next to the dorso-intercostal variety. It appears to be of rare occurrence in the earlier periods of life: the most remarkable case on record is perhaps the one related by Cotugno, in which the patient was only eleven years of age: males are more liable to the disease than females. There is little difference between the two sides of the body, in reference to the frequency of the attacks, but upon the whole the left has the preference. Out of 103 cases 46 were on the left, 43 on the right, and the remaining 14 were double. This view is confirmed by the observations of Romberg. In some instances M. Valleix thinks he has traced a decided connexion between the origin of the disease, and disturbance of the menstrual functions; it is very probable that this opinion is correct, but the table upon which it is founded (p. 573) is quite insufficient to bear out the conclusion. He has never met with a case in which the disease could be denominated syphilitic. Professor Romberg enumerates syphilis among the list of causes, but does not mention how many cases he has seen; indeed this remark applies to all his descriptions; there is no attempt any where at numerical deductions. These two authors are also at variance in regard to the metastatic origin of this affection; M. Valleix has never been able to trace it to such an occurrence; the German writer mentions several kinds of metastasis which may produce it. (Loc. cit. p. 63.)

The following are the points, in one or more of which a constant fixed pain is generally felt, and which are also painful upon pressure: 1. *In the loins*, but rarely. 2. *In the region of the hip; a*, near the *posterior superior spinous process of the ilium*, observed in all the cases but one, i. e., in 35 out of 36; *b*, about the middle of the crest of the ilium extending downwards a little upon the origin of the gluteus maximus, in 8 out of the 36 cases; *e*, at the upper part of the ischiatic notch, in 16 cases (Romberg also notices the fixed pain in this point); *d*, near the posterior edge of the great trochanter in 26 out of the 36 cases. 3. *On the thigh;* here it is much more rare to find isolated points, but in 4 cases M. Valleix detected three distinct parts in which there was pain on pressure, one near the tuberosity of the ischium, another in the middle of the thigh, and a third a little to the inside of the insertion of the biceps. 4. *At the knee;* in 17 out of the 36 cases the whole knee was painful, but even in these the suffering was greatest at the outside of the popliteal space, and at the head of the fibula; in 9 others these isolated points were alone affected. 5. *In the leg;* there are three principal localities of pain in this region; *a*, along the posterior edge of the fibula, observed in 28 out of the 36 cases; *b*, in the calf, over the septum, between the two heads of the gastrocnemii, in 15 cases; *c*, a little to the outside of the crest of the tibia in 5 cases. 6. *On the foot*, including the malleoli: this region was affected in 25 out of the 36 cases; in 8 the whole foot was painful, but in 7 of these there was also a very distinct point behind the external malleolus. The internal malleolus was affected in 3 others; and M. Valleix also detected painful points upon the toes. In 3 cases only was there any decided pain in the sole of the foot, and in these its precise locality could not be ascertained. We refer our readers to the slight anatomical sketch given above for an elucidation of the nature of these painful points.

*Lancinations* existed in all our author's cases, but not during the entire course of the disease, and in 3 out of 21 cases observed by M. Louis they were not manifested at all. This is an interesting feature in the disease, well worth the notice of future enquirers. *Cramp* was observed in 7 out of the 36 cases, and *convulsions of the limb* only in *one*, an old standing and very severe case. To this fact we request particular attention. Many patients complained of partial shiverings, and the sensation as of cold water running along the nerve. Professor Romberg affirms, that sciatica is seldom complicated with other forms; but in 12 out of 15 cases carefully examined by M. Valleix, neuralgic pains of various kinds were observed in different parts of the body, and he observes that when the principal disease was limited to one side, the complications were found on the same side of the body, when it was double they were also double, or existed only on the side which was most violently affected (p. 535.) M. Valleix has only met with one case in which the limb became atrophied. The attacks of pain frequently come on at night, especially upon first lying down in bed, being probably caused by the impression of cold from the sheets, not as M. Valleix imagines, from the diminished temperature of the air. Both the German and the French author insist upon the rareness of intermittence in this form of neuralgia. In reference to the *anatomical lesions* observed in sciatica, we shall be silent, merely referring our readers to the original (pp. 556 et seq.) for the celebrated case of Cotugno, and our author's very judicious remarks upon it.

*Diagnosis.* With proper attention, in most instances, this cannot be very difficult, yet disease of the hip-joint has been mistaken for it; a case of this kind is related by our author (p. 591), and is deserving of a careful perusal. We would refer also to an interesting case in which sciatica co-existed with articular rheumatism, the two alternating in violence and prominence of symptoms (p. 585 et seq.)

TREATMENT OF NEURALGIA. We now proceed to the consideration of the treatment of neuralgia, a subject which, notwithstanding its great importance, will detain us but a short time, because, excepting on one or two points, our author differs little from the opinions of preceding writers. We shall begin with the *internal* administration of remedies; and here, at the outset we would remark, that throughout the entire course of the work before us, M. Valleix does not once refer to the *purgative plan.* It is impossible that he can be ignorant of it, and equally so that he should be unaware of the success which has undoubtedly attended it in many cases, though it has failed in producing all the benefit that was once confidently anticipated from its employment. How the omission is to be explained we know not, but it is assuredly one of no small magnitude. Several years ago Sir Charles Bell reported in the Medical Gazette his success in the treatment of *tic* on this plan, the principal purgative being croton oil. He has noticed the subject at greater length in a late publication,* and Dr. Newbigging has recently published a report on the same subject.† Dr. Allnatt, in the little work on Tic Douloureux noticed in our last Number, has adopted the same mode of treatment, and, according to his report, with very great success. In some of these cases the croton oil would seem to have had some other (specific) effect, besides its general action as a purgative.

* Practical Essays, Edinb. 1841. † Edinb. Journal, Jan. 1841.

M. Valleix has no faith in the internal use of *narcotics* as a means of cure, having never met with a case in which the disease was removed by their employment. As palliatives they are of course extremely valuable, for in a disorder so painful, the most temporary relief is a matter of no little moment. The experience of practitioners in this country is, however, more favorable to this class of remedies, numerous cases being on record which have been cured by the exhibition of various sedative remedies, especially the *belladonna.* Our author speaks very doubtfully regarding the efficacy of the *sesquioxide of iron;* he does not seem to have employed it himself, and objects to the cases related by other observers, that the treatment has generally been too complex to allow of any decided opinion being formed. Unquestionably this objection applies to the majority of published cases, yet several are on record in which the beneficial effect of the medicine could hardly be called in question. He takes a more favorable view of the celebrated pills of Meglin (composed of hyoscyamus and oxide of zinc, āā gr. j. in each pill,) and believes that in many cases when the plan has failed, this event is to be attributed to the smallness of the dose, and the want of perseverance in the administration of the medicine. M. Méglin sometimes gave as many as from 36 to 48 pills a day without producing any bad effects, a somewhat wholesale mode of treatment!

When the paroxysms are decidedly periodical, the practitioner will do well to make trial either of the *quinine* or *arsenic;* remarkable success has been obtained from both.

We have yet to mention one other internal remedy which has been employed with singular advantage in the treatment of the sciatic form of neuralgia, we mean the *oil of turpentine.* This means of cure has been most successful in the hands of M. Martinet, and though, like all others, it will often fail, it is certainly one that deserves particular attention. Professor Romberg speaks highly of it; he has not observed that feeling of warmth extending from the intestinal canal to the nerves, in successful cases, which is insisted on by M. Martinet. He prefers the form of electuary, composed in the following manner:

℞ Ol. Tereb., ʒj.

Syrup. Aurant. vel. Mellis, ℥ij.

M. A tablespoonful twice a day.

If the taste be disagreeable to the patient, the following formula, recommended by Martinet, may be adopted:

℞ Ol. Tereb., ʒj.

Magnes. Calcin., ℈iiss.

Ol. Menth. gtt. viii.

M. A bolus of the size of a hazel nut to be taken in a wafer (*pain à chanter*), three times a day.

In reference to the employment of external remedies, our notice must be almost entirely confined to one plan of treatment, which in every case where it has been employed by our author has been productive of great alleviation in the symptoms, and which has often succeeded by itself in effecting a cure; we allude to the application of a succession of small blisters over the points in the course of the nerves which are *painful upon pressure.* Many of the instances recorded in the work before us, in which this remedy has rapidly removed the preexisting symptoms, are of the most striking nature, and we earnestly recommend their attentive perusal. The plan is not altogether original; Cotugno employed one very similar; but the idea of singling out the particular localities where there is either the constant dull pain, or which are painful upon pressure, is, we believe, to be attributed to M. Valleix alone. He considers this method decidedly preferable to the use of irritating ointments to the denuded surface of the skin, which is often productive of intolerable pain, and has frequently appeared rather to aggravate the symptoms than conduce to a cure. The *endermic* employment of morphia may be attempted with advantage in some cases, but it is a painful remedy. Dr. Basedow has procured much relief from careful bandaging of the affected limb. (Romberg, l. c. p. 70.)

The *actual cautery* has been employed with success, particularly by M. Jobert, but we perfectly agree with our author that it should never be employed until other means, and especially blisters, have been tried and found of no avail. The same remark applies to *section* of the nerve, and the removal of part of its substance, both of which so frequently disappoint the expectation of the practitioner and the hopes of the unfortunate patient. M. Valleix has seldom employed *electricity*, and never with success.

In the preceding pages we have endeavoured to give our readers a tolerably complete analysis of M. Valleix's very interesting book, and we again heartily recommend a perusal of the original work. We do not feel ourselves authorized to regard his opinions as clearly established, but the amount of evidence which he has brought forwards in support of them is surely enough to demand attention on the part of other practitioners. If his views be correct, they afford us a method of investigation of the highest value, both as regards our diagnosis and prognosis of these protean maladies, and may possibly at some future period conduce to the formation of something like a satisfactory theory of their nature. The work would bear condensation, and would be considerably improved by the omission of many needless repetitions, especially the double descriptions of each form of neuralgia, of which we cannot perceive that the advantage does in any way compensate for the tedium.

We cannot bring this article to a conclusion without briefly noticing the opinions of Dr. M. Hall upon the subject of neuralgia, as developed in his recent work on the Diseases and Derangements of the Nervous System. Neuralgia is included by Dr. Hall in the list of *cerebral diseases*, under the head "Augmented action of the sentient nerves." (§ 1485.) *Spasmodic tic* finds its place among the diseases of the *reflex* or *motor nerves* of the *true spinal system*. (§ 415.) Are these then two separate diseases? We think not; and we do not apprehend that Dr. Hall regards them as such, though it is not very easy to understand his views. Assuredly the cases recorded by him as instances of *spasmodic tic* appear to be wanting in the most essential symptom of neuralgia, viz. pain, (vid. pp. 343-7); but under the same head he makes the following remark: "In one case, exposure to severe cold induced numbness of the face, which gradually passed into a terrible *tic douloureux*." (§ 1753.)

Again, in his description of neuralgia, after describing the nature of the paroxysms of *tic douloureux* as being "sudden, irregular in their occurrence, frequently more or less transient or momentary, induced, when in the face, by the act of eating, or talking, or by the contact of external bodies with the acutely sensitive extremities of the nerves," he observes, "This disease is distinguished by that which the term *tic* means originally: viz. by a sudden contraction of several muscles, with distortion of of the face. Its seat is various—in different parts of the face, of the limbs, and of other parts of the surface of the body." (§ 1500-1.)*

This last sentence may, perhaps, afford an explanation of the apparent difficulty. If Dr. Hall regards *spasm* as the characteristic symptom of the affection, we can understand why he should include under the same appellation cases in which pain did not exist, or at any rate was not prominent, (for we are surely at liberty to suppose that this was the state of matters in the instances above referred to, seeing that no reference whatever is made to it.) But is this view of the matter warranted by facts? Is it true that *spasm* is the chief characteristic of neuralgia? Dr. Rowland, in his excellent work on this disease, observes—"The addition of *spasmodica* [to the term neuralgia] is objectionable, because it refers either to an obscure theory of the disease, or to a symptom of *comparatively rare occurrence*, namely, the convulsive motion of the facial muscles during the paroxysms." (Rowland on Neuralgia, p. 98.) M. Valleix out of fourteen cases of facial neuralgia, carefully examined by himself, only met with *four* in which this symptom existed, and in these the spasms were so slight that there was no distortion of the face. (p. 93.) In all the recorded cases which he scrutinized he could only find sixteen in which they were observed. It will be remembered that the whole number of cases which he analysed amounted to fifty-five. In the other varieties the occurrence of this symptom is still less frequent; we doubt if it ever exists when the disease is confined to the trunk, and M. Valleix has only met with *one* case of violent involuntary contractions of the muscles out of thirty-six affected with sciatica. We should like to be informed upon how many observations Dr. Hall has founded his assertion, which is so greatly at variance with the experience of other investigators.

But while we thus differ from him upon this point, we have great pleasure in bearing our testimony to the value of his researches as affording an explanation of the method in which these spasms are occasionally produced, and would hazard the opinion, (which by the way is probably his own, though we do not find it expressed in so many words,) that their presence or absence depend upon the fact of the *incident* filaments being involved in the affection, or the disease being strictly confined to the mere *central sensitive fibres*. If this idea be correct, it will throw great light upon what might otherwise be regarded as a singular anomaly, and will not be without use in reference to the prognosis.

* We quote these passages in Dr. Hall's own words, in order to avoid as much as possible all risk of misapprehension.

